# Comparison between Variable and Conventional Volume-Controlled Ventilation on Cardiorespiratory Parameters in Experimental Emphysema

**DOI:** 10.3389/fphys.2016.00277

**Published:** 2016-06-30

**Authors:** Isabela Henriques, Gisele A. Padilha, Robert Huhle, Caio Wierzchon, Paulo J. B. Miranda, Isalira P. Ramos, Nazareth Rocha, Fernanda F. Cruz, Raquel S. Santos, Milena V. de Oliveira, Sergio A. Souza, Regina C. Goldenberg, Ronir R. Luiz, Paolo Pelosi, Marcelo G. de Abreu, Pedro L. Silva, Patricia R. M. Rocco

**Affiliations:** ^1^Laboratory of Pulmonary Investigation, Carlos Chagas Filho Biophysics Institute, Federal University of Rio de JaneiroRio de Janeiro, Brazil; ^2^Pulmonary Engineering Group, Department of Anesthesiology and Intensive Care Therapy, University Hospital Carl Gustav Carus, Technische Universität DresdenDresden, Germany; ^3^Laboratory of Molecular and Cellular Cardiology, Carlos Chagas Filho Biophysics Institute, Federal University of Rio de JaneiroRio de Janeiro, Brazil; ^4^National Center for Structural Biology and Bioimaging, Federal University of Rio de JaneiroRio de Janeiro, Brazil; ^5^Department of Physiology and Pharmacology, Biomedical Institute, Fluminense Federal UniversityNiterói, Brazil; ^6^Nuclear Medicine Service, Clementino Fraga Filho University Hospital, Federal University of Rio de JaneiroRio de Janeiro, Brazil; ^7^Institute of Public Health Studies, Federal University of Rio de JaneiroRio de Janeiro, Brazil; ^8^Department of Surgical Sciences and Integrated Diagnostics, IRCCS AOU San Martino IST, University of GenoaGenoa, Italy

**Keywords:** echocardiography, elastic fiber, surfactant protein-D, elastance, alveolar hyperinflation

## Abstract

Emphysema is characterized by loss of lung tissue elasticity and destruction of structures supporting alveoli and capillaries. The impact of mechanical ventilation strategies on ventilator-induced lung injury (VILI) in emphysema is poorly defined. New ventilator strategies should be developed to minimize VILI in emphysema. The present study was divided into two protocols: (1) characterization of an elastase-induced emphysema model in rats and identification of the time point of greatest cardiorespiratory impairment, defined as a high specific lung elastance associated with large right ventricular end-diastolic area; and (2) comparison between variable (VV) and conventional volume-controlled ventilation (VCV) on lung mechanics and morphometry, biological markers, and cardiac function at that time point. In the first protocol, Wistar rats (*n* = 62) received saline (SAL) or porcine pancreatic elastase (ELA) intratracheally once weekly for 4 weeks, respectively. Evaluations were performed 1, 3, 5, or 8 weeks after the last intratracheal instillation of saline or elastase. After identifying the time point of greatest cardiorespiratory impairment, an additional 32 Wistar rats were randomized into the SAL and ELA groups and then ventilated with VV or VCV (*n* = 8/group) [tidal volume (V_T_) = 6 mL/kg, positive end-expiratory pressure (PEEP) = 3 cmH_2_O, fraction of inspired oxygen (FiO_2_) = 0.4] for 2 h. VV was applied on a breath-to-breath basis as a sequence of randomly generated V_T_ values (mean V_T_ = 6 mL/kg), with a 30% coefficient of variation. Non-ventilated (NV) SAL and ELA animals were used for molecular biology analysis. The time point of greatest cardiorespiratory impairment, was observed 5 weeks after the last elastase instillation. At this time point, interleukin (IL)-6, cytokine-induced neutrophil chemoattractant (CINC)-1, amphiregulin, angiopoietin (Ang)-2, and vascular endothelial growth factor (VEGF) mRNA levels were higher in ELA compared to SAL. In ELA animals, VV reduced respiratory system elastance, alveolar collapse, and hyperinflation compared to VCV, without significant differences in gas exchange, but increased right ventricular diastolic area. Interleukin-6 mRNA expression was higher in VCV and VV than NV, while surfactant protein-D was increased in VV compared to NV. In conclusion, VV improved lung function and morphology and reduced VILI, but impaired right cardiac function in this model of elastase induced-emphysema.

## Introduction

Chronic obstructive pulmonary disease (COPD) is characterized by persistent airflow limitation due to an enhanced chronic inflammatory response (GOLD, 2015^1^), and can lead to respiratory failure and the need for ventilator support (MacIntyre and Huang, 2008). Although the use of noninvasive positive-pressure ventilation for COPD is increasingly popular and associated with shorter length of hospital stay, lower mortality rates, and lower costs, severe cases still require invasive mechanical ventilation (Stefan et al., 2015a,b). In patients with COPD, invasive volume-controlled (VCV) or pressure-controlled mechanical ventilation may exacerbate preexisting lung damage as a result of time-constant inhomogeneity, which predisposes to delayed inflation of some lung areas and overdistension of other regions (Laghi et al., 2001; MacIntyre and Huang, 2008); this, in turn, may lead to ventilator-induced lung injury (VILI) and a negative impact on right ventricular function (Vieillard-Baron et al., 1999; Wrobel et al., 2015). Therefore, a mechanical ventilation strategy able to promote a more homogeneous distribution of aeration without affecting cardiac function could be a useful therapeutic option for patients with COPD. Variable ventilation (VV) can improve surfactant production (Arold et al., 2009), maintain airway patency (Suki et al., 1994), and reduce cyclic closing/reopening, thus decreasing shear stress, inflammation, and epithelial cell damage (Thammanomai et al., 2013). In addition, VV promotes lung functional benefits in experimental acute respiratory distress syndrome (ARDS) (Spieth et al., 2009b; Ruth Graham et al., 2011), severe bronchospasm (Mutch et al., 2007), and prolonged anesthesia (Mutch et al., 2000).

The effects of mechanical ventilation on VILI in patients with COPD were derived from pre-clinical and clinical studies in ARDS. To date, however, no study has evaluated the impact of VCV and VV on VILI in experimental or clinical emphysema.

Since emphysema is characterized by loss of lung tissue elasticity and destruction of structures supporting alveoli and capillaries, we hypothesized that VV might reduce lung hyperinflation and atelectasis, thus improving pulmonary function, with less epithelial and endothelial cell damage and inflammation. The present study was divided into two protocols: (1) characterization of an elastase-induced emphysema model in rats, with identification of the time point of greatest cardiorespiratory impairment, defined as a high specific lung elastance associated with large right ventricular end-diastolic area; and (2) comparison, at that time point, of the effects of VV vs. VCV on gas exchange, lung mechanics and histology, right ventricular function, biological markers associated with inflammation, damage inflicted to alveolar epithelial and endothelial cells, and alveolar stretch.

## Materials and methods

This study was approved by the Ethics Committee of the Health Sciences Center, Federal University of Rio de Janeiro. All animals received humane care in compliance with the Principles of Laboratory Animal Care formulated by the National Society for Medical Research and the U.S. National Academy of Sciences *Guide for the Care and Use of Laboratory Animals.*

### Animal preparation and experimental protocol

#### First protocol: characterization of elastase-induced emphysema model

Sixty-two Wistar rats were randomly assigned across two groups: SAL and ELA. In ELA group, rats received porcine pancreatic elastase (2 IU in 0.1 ml of saline solution, Sigma Chemical Co., St. Louis, MO, USA) intratracheally, once weekly for 4 weeks. SAL animals received saline solution only via the same route and dosage schedule. Before each intratracheal instillation, animals were premedicated with intraperitoneal diazepam (10 mg/kg, Compaz®, Cristália, Itapira, SP, Brazil) and anesthetized with 1.5–2.0% isoflurane (Cristália, SP, Brazil) by mask.

Evaluations were performed 1, 3, 5, or 8 weeks after the last intratracheal instillation of saline or porcine pancreatic elastase (Figure S1). Briefly, animals were anesthetized with intraperitoneal diazepam (10 mg/kg, Compaz®, Cristália, Itapira, SP, Brazil), ketamine (50–100 mg/kg, Ketamin-S+®, Cristália, Itapira, SP, Brazil), and midazolam (2 mg/kg, Dormicum®, União Química, São Paulo, SP, Brazil), tracheotomized, paralyzed with pancuronium bromide (2 mg/kg i.v., Cristália, Itapira, SP, Brazil), and mechanically ventilated (Servo-i, MAQUET, Solna, Sweden) in VCV mode, using the following parameters: tidal volume (V_T_) = 6 mL/kg, respiratory rate (RR) = 80 breaths/min, fraction of inspired oxygen (FiO_2_) = 0.4, and positive end-expiratory pressure (PEEP) = 3 cmH_2_O. During ventilation, intravenous fluids were administered to maintain a mean arterial pressure (MAP) ≥70 mmHg and midazolam plus ketamine were administered to maintain sedation. Fifty-six rats were used to evaluate lung mechanics and morphometry, end-expiratory lung volume (EELV), biological markers associated with inflammation (interleukin [IL]-6, cytokine-induced neutrophil chemoattractant [CINC]−1), alveolar stretch (amphiregulin), type II epithelial cell mechanotransduction (surfactant protein [SP]-D), and endothelial cell damage (angiopoietin [Ang]-2 and vascular endothelial growth factor [VEGF]), as well as echocardiographic parameters (*n* = 7 at each time point in SAL and ELA groups). In six animals (*n* = 3/group), a computed tomography scan of the lungs was performed at 5 weeks.

#### Second protocol: comparison between variable and conventional volume-controlled ventilation

After identifying the time point of greatest cardiorespiratory impairment, an additional 32 Wistar rats were randomized into the SAL and ELA groups, as previously described. Animals were premedicated and anesthetized as described for the first protocol. An intravenous catheter (Jelco 24G) was inserted into the tail vein for continuous infusion of midazolam (2 mg/kg/h), ketamine (50 mg/kg/h), and Ringer's lactate (7 mL/kg/h, B. Braun, Crissier, Switzerland). Anesthetized animals were kept in the dorsal recumbent position and tracheotomized via a midline neck incision after subcutaneous injection of 2% lidocaine (Cristália, Itapira, SP, Brazil). The right internal carotid artery was cannulated (18 G, Arrow International, USA) for blood sampling and MAP measurement. Heart rate (HR), MAP, and rectal temperature were continuously recorded (Networked Multiparameter Veterinary Monitor LifeWindow 6000 V, Digicare Animal Health, Florida, USA). Body temperature was maintained at 37.5 ± 1°C using a heating bed. Gelafundin® (B. Braun, São Gonçalo, RJ, Brazil) was administered intravenously in 0.5-mL increments to keep MAP ≥ 70 mmHg. Animals were paralyzed by intravenous administration of pancuronium bromide (2 mg/kg, Cristália, Itapira, SP, Brazil) and mechanically ventilated (Inspira, Harvard Apparatus, Holliston, Massachusetts, USA) in VCV mode with V_T_ = 6 mL/kg, RR adjusted to maintain arterial pHa in the 7.35–7.45 range, Fio_2_ = 0.4, and zero end-expiratory pressure (ZEEP). After hemodynamic stabilization, respiratory system mechanics, and arterial blood gases (Radiometer ABL80 FLEX, Copenhagen NV, Denmark) were measured (Baseline ZEEP). PEEP was then increased to 3 cmH_2_O and, after 5 min, respiratory system mechanics, arterial blood gases, and echocardiographic parameters were analyzed (Baseline PEEP). Following this step, SAL and ELA animals were randomized to VV or VCV (*n* = 8/group). VV was applied on a breath-to-breath basis as a sequence of randomly generated V_T_ values (Gaussian distribution, *n* = 1200; mean V_T_ = 6 mL/kg), with a 30% coefficient of variation (nVentInspira, Dresden, Germany), for 2 h (Huhle et al., 2014). At the end of the experiment, echocardiographic parameters, respiratory system mechanics, and arterial blood gases were analyzed at each ventilator setting. Animals were then euthanized via overdose of intravenous sodium thiopental (50 mg/kg, Cristália, Itapira, SP, Brazil) and their lungs extracted at PEEP = 3 cmH_2_O for measurement of EELV, as well as lung histological and molecular biology analyses (Figure S2).

### Echocardiography

#### First protocol

Echocardiographic measurements were obtained at baseline and 1, 3, 5, and 8 weeks after the last intratracheal instillation of SAL or PPE. Right ventricular (RV) end-diastolic area, pulmonary artery acceleration time (PAT), pulmonary artery ejection time (PET), and their ratio (PAT/PET) were used as indirect indexes of pulmonary arterial hypertension.

#### Second protocol

Echocardiographic parameters were measured before and 2 h after mechanical ventilation (VV or VCV) in the SAL and ELA groups. In addition to the aforementioned parameters associated with right ventricular impairment, left ventricle diastolic area, ejection fraction, and fractional shortening were computed, as mechanical ventilation may also affect left systolic function.

#### Technique

Animals were placed in the dorsal recumbent position and the precordial region was shaved. Transthoracic echocardiography was performed by an expert (NR), using a 10-MHz probe (Esaote model, CarisPlus, Firenze, Italy). Images were obtained from the subcostal and parasternal views. Short-axis two-dimensional views of the left and right ventricles were acquired at the level of the left ventricular papillary muscles to measure left and right ventricular end-diastolic area (LV and RV area, respectively). The left ventricular ejection fraction and fractional shortening were calculated in one-dimensional mode analysis of the left ventricle guided by the parasternal short-axis view. Pulsed-wave Doppler was used to measure PAT, PET, and the PAT/PET ratio. The diameter of the right atrium and inferior vena cava were assessed from the subcostal view. Measurements were obtained in accordance with American Society of Echocardiography Guidelines (Thibault et al., 2010; Lang et al., 2015).

### Computed tomography

#### First protocol

Computed tomography (CT) scans were performed to evaluate the presence of emphysematous areas. CT was performed in SAL and ELA animals at the time point of greatest cardiorespiratory impairment (5 weeks after the last elastase administration) with an Optima 560 PET/CT scanner (GE Healthcare, Boston, USA). The acquisition protocol was based on helical CT with axial slices of 0.625 mm (16 × 0.625 mm) thickness and 48 images, with a beam collimation of 10.0 mm and a DFOV of 10 cm. The X-ray tube was set to 120 kV and 80 mA. The total time for each scan was 12 s. Hounsfield units (HU) were analyzed in both lungs, at the bifurcation of pulmonary arteries, in the Onis 2.5 software environment (DigitalCore, Co. Ltd., Tokyo, Japan).

### Lung mechanics

#### First protocol

Airflow, volume, and airway and esophageal pressures were measured (Riva et al., 2008), and lung mechanics were analyzed by the end-inflation occlusion method (Bates et al., 1985). Static lung elastance (Est,L) was calculated by dividing the difference between respiratory system and chest wall elastic pressure (Pel,L) by V_T_. Since EELV may change due to emphysema, Est,L was normalized by EELV, which is equal to specific lung elastance (EL,spec).

#### Second protocol

Airflow, volume, and airway pressure were continuously recorded. Elastance (E,_RS_) and resistance (R,_RS_) were calculated based on the equation of motion (Uhlig et al., 2014). Volume-independent elastance (E1,_RS_) and volume-dependent elastance (E2,_RS_) were calculated on a cycle-by-cycle basis (Carvalho et al., 2013). The partition model of respiratory system elastance allows calculation of the E,_RS_ non-linearity index (%E2), which quantifies the concavity of the dynamic pressure-volume (PV) curve, thus enabling identification of tidal recruitment/overdistension, which can be present in this emphysema model.

#### Technique

All parameters were recorded with a computer running custom software written in LabVIEW® (National Instruments; Austin, Texas, USA) (Silva et al., 2013). All signals were amplified in a three-channel signal conditioner (TAM-D HSE Plugsys Transducers Amplifiers, Module Type 705/2, Harvard Apparatus, Holliston, Massachusetts, USA) and sampled at 200 Hz with a 12-bit analog-to-digital converter (National Instruments; Austin, Texas, USA).

### End-expiratory lung volume measurement

#### First and second protocols

EELV was determined as previously described (Scherle, 1970) to identify the resulting lung volume at end-expiration, as emphysema is characterized by destruction of alveolar septa with hyperinflated areas. Briefly, a jar containing sufficient saline solution with a surplus weight submerged was placed on a common laboratory scale, which was subsequently tared to zero. The lungs were fixed to a laboratory stand by means of a thread with the surplus weight and completely submerged in the saline solution. The liquid displaced by the submerged lungs adds correspondingly to the weight on the scale. Because the specific gravity of saline differs no more than 2–3% from 1 g/cm^3^, the volume of the organ may be expressed directly by the weight gain registered on the scale.

### Histology

#### First protocol

Lung morphometry was analyzed in SAL and ELA animals to characterize the model of emphysema.

#### Second protocol

Lung morphometry was evaluated in animals ventilated with VCV and VV, as well as in non-ventilated (NV) animals (SAL and ELA), to analyze the impact of different ventilator strategies on lung parenchyma.

#### Technique

Morphometric analysis was performed in lungs excised at end-expiration (PEEP = 3 cmH_2_O). Immediately after removal, the left lung was flash-frozen by immersion in liquid nitrogen, fixed with Carnoy's solution, and paraffin-embedded. Sections (4 μm thick) were cut and stained with hematoxylin-eosin. Investigators (CW and VLC) blinded to the origin of the material performed the microscopic examination. Morphometric analysis was done using an integrating eyepiece with a coherent system made of a 100-point grid consisting of 50 lines of known length, coupled to a conventional light microscope (Axioplan, Zeiss, Oberkochen, Germany). The volume fraction of collapsed and normal pulmonary areas and the fraction of the lung occupied by large-volume gas-exchanging air spaces (hyperinflated structures with a morphology distinct from that of alveoli and wider than 120 μm) were determined by the point-counting technique (Weibel, 1990), at a magnification of × 200 across 10 random, noncoincident microscopic fields. Briefly, points falling on collapsed or normal pulmonary areas or hyperinflated alveoli were counted and divided by the total number of points in each microscopic field. Lung tissue distortion was assessed by measuring the mean linear intercept between alveolar walls (Lm) at a magnification of × 400 (Weibel, 1990). In the first protocol, collagen and elastic fibers (stained with the Picrosirius-polarization method and Weigert's resorcin fuchsin modified with oxidation, respectively) were quantified in alveolar septa at × 400 magnification (Antunes et al., 2014). The area occupied by fibers was determined by digital densitometric recognition in the Image-Pro Plus 7.1 for Windows software environment (Media Cybernetics, Silver Spring, MD, USA) and divided by the area of each studied septum. Results were expressed as the fractional area occupied by elastic and collagen fibers in the alveolar septa. Bronchi and blood vessels were excluded from the measurements.

### Molecular biology

#### First and second protocols

Quantitative real-time reverse transcription polymerase chain reaction (RT-PCR) was performed to assess IL-6, CINC-1, amphiregulin, SP-D, Ang-2, and VEGF. In the first protocol, these analyses were performed at the time point of greatest cardiorespiratory impairment. Central slices of the right lung were cut, collected in cryotubes, flash-frozen by immersion in liquid nitrogen, and stored at −80°C. Total RNA was extracted from frozen tissues using the RNeasy Plus Mini Kit (Qiagen, Hilden, Germany) following the manufacturer's recommendations. RNA concentration was measured by spectrophotometry in a Nanodrop ND-1000 system (ThermoScientific, Wilmington, DE, USA). First-strand cDNA was synthesized from total RNA using a Quantitec reverse transcription kit (Qiagen, Hilden, Germany). The primers used are described in the online supplement (Table S1). Relative mRNA levels were measured with a SYBR green detection system in ABI 7500 real-time PCR (Applied Biosystems, Foster City, California, USA). Samples were run in triplicate. For each sample, the expression of each gene was normalized to the acidic ribosomal phosphoprotein P0 (*36B4*) housekeeping gene and expressed as fold changes relative to SAL animals (first protocol) or to non-ventilated (NV) animals (SAL and ELA—second protocol), using the 2^−ΔΔCt^ method, where ΔCt = Ct_reference gene_ − Ct_target gene_.

### Statistical analysis

The number of animals per group was based on pilot studies of the elastase instillation model of emphysema. A sample size of eight animals per group (providing for one animal as dropout) would provide the appropriate power (1 − β = 0.8) to identify significant (α = 0.05) differences in dynamic respiratory system elastance between VCV and VV, taking into account an effect size *d* = 1.57, a two-sided test, and a sample size ratio = 1 (G^*^Power 3.1.9.2, University of Düsseldorf, Germany).

In the first protocol, one-way ANOVA followed by Bonferroni's *post-hoc* test was used to evaluate differences among SAL or ELA group at different time points. As no significant differences were observed among SAL groups at each time point, all data were into a single SAL group. At each time point, SAL was compared to ELA using Student's *t*-test followed by Bonferroni adjustment, with α adjusted to four comparisons (0.05/4 = 0.0125).

In the second protocol, a paired *t*-test was used to compare data between Baseline PEEP and End. Two-way repeated-measures ANOVA followed by Bonferroni's *post-hoc* test was used to compare cardiorespiratory function parameters between SAL and ELA groups ventilated with VV and VCV. One-way ANOVA followed by Bonferroni's *post-hoc* test was used to compare lung morphometry between NV, VV, and VCV in the SAL and ELA groups. Molecular biology analyses were performed using the Kruskal–Wallis test followed by Dunn's multiple comparison test within the SAL (NV, VV, VCV) and ELA (NV, VV, VCV) groups. Parametric data were expressed as mean ± standard deviation (SD) and nonparametric data as median (interquartile range). All tests were performed using the GraphPad Prism v6.07 statistical software package (GraphPad Software, La Jolla, California, USA).

## Results

### First protocol: characterization of elastase-induced emphysema in rats

**Figure 1:**
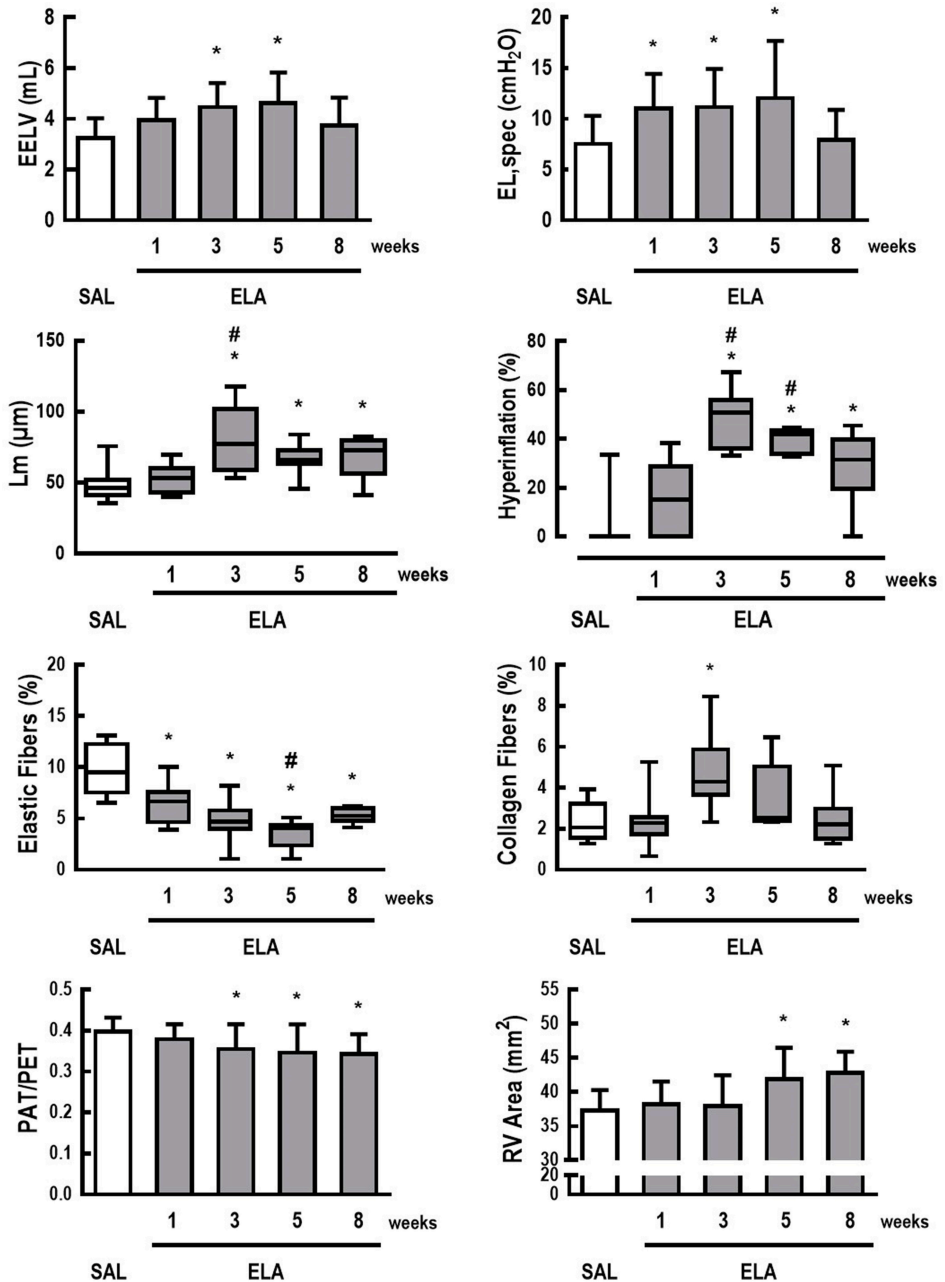
**Characterization of elastase-induced emphysema model in rats**. Data are presented as means + standard deviation (SD) or as box plots of median and interquartile range (whiskers indicate the 10th and 90th percentiles), and refer to 7 rats in each ELA group and 28 rats in the SAL group. EELV, end-expiratory lung volume; EL,spec, specific lung elastance; Lm, mean linear intercept between alveolar walls; hyperinflation, fraction area of hyperinflated areas; Elastic Fibers, fraction area of elastic fibers; Collagen Fibers, fraction area of collagen fibers; PAT/PET, ratio between pulmonary artery systolic acceleration time (PAT) and pulmonary artery systolic ejection time (PET) on pulsed-wave doppler (A.U.); RV Area, right ventricular end-diastolic area (mm^2^). *Significantly different from SAL group (*p* < 0.0125). ^#^Significantly different from ELA at 1 week (*p* < 0.0125).

**Figure 2:**
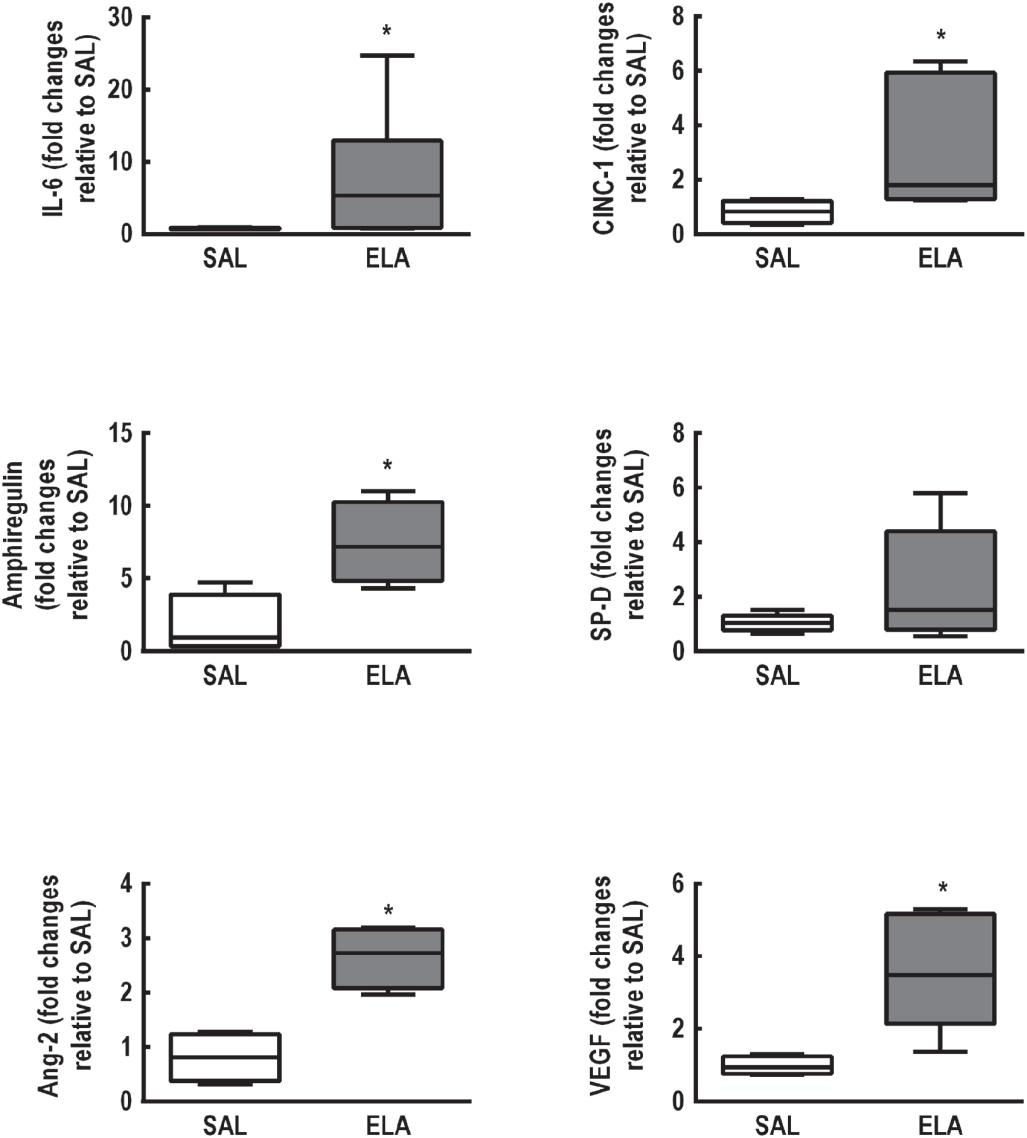
**Real-time polymerase chain reaction analysis of biological markers associated with inflammation (interleukin [IL]-6, cytokine-induced neutrophil chemoattractant [CINC]-1), alveolar stretch (amphiregulin), type II epithelial cell mechanotransduction (surfactant protein [SP]-D), and endothelial cell damage (angiopoietin [Ang]-2, vascular endothelial growth factor [VEGF])**. SAL: animals that received saline and were analyzed 5 weeks after the last saline endotracheal instillation; ELA: animals that received elastase and were analyzed 5 weeks after the last elastase endotracheal instillation. Data are presented as box plots of median and interquartile range (whiskers indicate the 10th and 90th percentiles), and refer to seven animals in the SAL and ELA groups. Relative gene expression was calculated as a ratio of the average gene expression levels compared with the reference gene (36B4) and expressed as fold change relative to SAL. *Significantly different from SAL group (*p* < 0.05).

Figure 1 depicts the time course of EELV, EL,spec, Lm, fraction area of hyperinflated alveoli (hyperinflation), percentage of elastic and collagen fibers, PAT/PET ratio, and RV area in the ELA group. Even though lung mechanics and collagen fiber content tended to return to SAL values, hyperinflation and Lm remained higher than in SAL animals, associated with elastolysis. Additionally, a continuous deterioration of cardiovascular function was observed, reaching significance 3–5 weeks after the last dose of elastase, with no subsequent recovery. The time point of greatest cardiorespiratory impairment, defined as a high EL,spec associated with large RV area, was observed 5 weeks after the last elastase instillation. At this time point, mRNA expressions of IL-6 [median (interquartile range): ELA, 5.33 (0.82–12.89) vs. SAL, 0.64 (0.62–0.88)], CINC-1 [ELA, 1.79 (1.28–5.92) vs. SAL, 0.83 (0.42–1.21)], amphiregulin [ELA, 7.18 (4.82–10.27) vs. SAL, 0.94 (0.36–3.90)], Ang-2 [ELA, 2.72 (2.08–3.15) vs. SAL, 0.81 (0.38–1.23)], and VEGF [ELA, 3.47 (2.14–5.16) vs. SAL, 0.95 (0.76–1.24)] were higher in ELA compared to SAL, with no significant changes in SP-D expression (Figure 2). Figure S3 depicts CT images from SAL and ELA groups. SAL animals showed densities from −543 to −496 HU and −608 to −525 HU for the right and left lungs, respectively. On the other hand, ELA animals showed densities from −914 to −466 HU and −929 to −842 HU for the right and left lungs, respectively.

### Second protocol: comparison between variable and conventional volume-controlled ventilation

MAP decreased over time in SAL and ELA animals alike and was lower in VV compared to VCV, but remained ≥ 70 mmHg (Figure S4). At Baseline ZEEP, respiratory variables and arterial blood gases did not differ among groups (Table S2).

Table 1 shows respiratory and gas-exchange variables at Baseline PEEP and End. V_T_, V'_E_, PEEP, and intrinsic positive end-expiratory pressure (PEEPi) levels were comparable among groups, whereas the coefficient of variation in V_T_ was higher in VV compared to VCV (VV: 28.0 ± 2.1 vs. VCV: 2.2 ± 0.9, *p* < 0.0001). At End, E,_RS_ was lower in VV than VCV in both the SAL and ELA groups (VV: 2.5 ± 0.4 vs. VCV: 3.6 ± 0.8, *p* = 0.0003). In ELA, E2,_RS_ was reduced in VV compared to VCV (VV: 0.39 ± 0.20 vs. VCV: 0.81 ± 0.25, *p* = 0.021). The arterial partial pressure of oxygen (PaO_2_) increased over time, with no significant differences in arterial pH, arterial partial pressure of carbon dioxide (PaCO_2_), or bicarbonate (HCO${}_{3}^{-}$) between VCV and VV in either group (SAL and ELA), as shown in Table 1.

**Table 1:** **Respiratory and blood gas exchange parameters at Baseline PEEP and End**.

		**SAL**		**ELA**	
		**VCV**	**VV**	**VCV**	**VV**
V_T_	Baseline PEEP	6.0 ± 0.0	6.0 ± 0.0	6.0 ± 0.0	6.0 ± 0.0
(mL/kg)	End	6.0 ± 0.1	6.1 ± 0.1	6.0 ± 0.0	6.1 ± 0.1
CV of V_T_	Baseline PEEP	2.0 ± 0.3	2.0 ± 0.2	2.1 ± 0.4	2.0 ± 0.3
(%)	End	2.1 ± 0.4	28.2 ± 0.6^#^, ^&^	2.2 ± 0.9	28.0 ± 2.1^‡^, ^&^
V'_E_	Baseline PEEP	147.8 ± 4.6	145.5 ± 6.1	144.5 ± 3.5	148.9 ± 1.9
(mL/min)	End	146.2 ± 12.1	145.6 ± 5.3	144.7 ± 9.6	149.3 ± 7.5
E,_RS_	Baseline PEEP	3.3 ± 0.4	3.5 ± 0.3	3.7 ± 0.8	3.5 ± 0.7
(cmH_2_O/mL)	End	3.6 ± 0.1	2.3 ± 0.4^#^, ^&^	3.6 ± 0.8	2.5 ± 0.4^‡^, ^&^
E1,_RS_	Baseline PEEP	3.0 ± 0.7	2.7 ± 0.4	2.5 ± 0.5	2.4 ± 0.5
(cmH_2_O/mL)	End	3.3 ± 1.1	2.4 ± 0.6^&^	2.7 ± 0.6	2.2 ± 0.5
E2,_RS_	Baseline PEEP	0.32 ± 0.38	0.48 ± 0.21	0.71 ± 0.21	0.76 ± 0.42
(cmH_2_O/mL)	End	0.43 ± 0.39	0.19 ± 0.13^&^	0.81 ± 0.25	0.39 ± 0.20^‡^, ^&^
%E2	Baseline PEEP	29.8 ± 37.6	48.9 ± 16.4	62.3 ± 7.8	62.1 ± 14.8
	End	36.9 ± 37.1	32.4 ± 19.1^&^	63.1 ± 11.0	49.4 ± 19.3^&^
R	Baseline PEEP	0.22 ± 0.11	0.24 ± 0.11	0.27 ± 0.13	0.20 ± 0.07
(cmH_2_O/mL/s)	End	0.22 ± 0.11	0.20 ± 0.10	0.23 ± 0.09	0.21 ± 0.10
PEEP	Baseline PEEP	3.0 ± 0.1	3.1 ± 0.1	3.0 ± 0.1	3.1 ± 0.1
(cmH_2_O)	End	3.0 ± 0.1	3.1 ± 0.1	3.0 ± 0.2	3.1 ± 0.1
PEEPi	Baseline PEEP	0.4 ± 0.1	0.5 ± 0.5	0.5 ± 0.2	0.4 ± 0.1
(cmH_2_O)	End	0.4 ± 0.1	0.4 ± 0.1	0.4 ± 0.1	0.4 ± 0.1
pHa	Baseline PEEP	7.4 ± 0.0	7.4 ± 0.0	7.4 ± 0.1	7.4 ± 0.1
	End	7.4 ± 0.1	7.4 ± 0.0	7.3 ± 0.1	7.4 ± 0.1
PaO_2_	Baseline PEEP	108 ± 27	125 ± 28	111 ± 31	121 ± 22
(mmHg)	End	166 ± 48^&^	191 ± 51^&^	161 ± 51^&^	199 ± 34^&^
PaCO_2_	Baseline PEEP	37.6 ± 4.1	34.5 ± 6.7	35.2 ± 9.3	37.5 ± 10.3
(mmHg)	End	35.0 ± 6.8	35.1 ± 2.6	40.1 ± 7.1	33.9 ± 9.0
HCO_3_	Baseline PEEP	23.6 ± 1.6	23.2 ± 2.3	21.8 ± 4.3	22.6 ± 2.3
(mEq/L)	End	22.4 ± 2.9	22.6 ± 1.9	22.0 ± 2.0	20.1 ± 2.7

Values are mean ± standard deviation (SD) of eight animals in each group. SAL: animals that received saline and were analyzed 5 weeks after the last saline endotracheal instillation; ELA: animals that received elastase and were analyzed 5 weeks after the last elastase endotracheal instillation; VCV: volume-controlled ventilation; VV: variable ventilation; V_T_: tidal volume; CV of V_T_: coefficient of variation of tidal volume; V'_E_: minute ventilation; E,_RS_: dynamic respiratory system elastance; E1,_RS_: volume-independent elastance; E2,_RS_: volume-dependent elastance; %E2: E,_RS_ non-linearity index; R: airway resistance; PEEP: positive end-expiratory pressure; PEEPi: intrinsic positive end-expiratory pressure; pHa: arterial pH; PaCO_2_: arterial carbon dioxide partial pressure; PaO_2_: arterial oxygen partial pressure; HCO_3_: bicarbonate. Comparisons were performed by two-way repeated-measures ANOVA followed by Bonferroni's post-hoc test (p < 0.05). Paired t-tests were used to compare Baseline PEEP and End.

# Significantly different from SAL-VCV (p < 0.05).

‡ Significantly different from ELA-VCV (p < 0.05).

& Significantly different from Baseline PEEP (p < 0.05).

In the ELA group, the fraction area of normal alveoli was increased, while areas of alveolar collapse and hyperinflation were decreased in both VCV and VV compared to NV. The fraction areas of alveolar collapse and hyperinflation were smaller in VV than VCV (alveolar collapse: VV, 5.3 ± 2.6 vs. VCV, 11.2 ± 6.8, *p* = 0.028; hyperinflation: VV, 3.5 ± 2.6 vs. VCV, 14.0 ± 7.6, *p* = 0.0008). EELV was higher in ELA compared to SAL groups, regardless of ventilation mode (Table 2).

**Table 2:** **Lung morphometry in mechanically ventilated animals**.

	**SAL**			**ELA**		
	**NV**	**VCV**	**VV**	**NV**	**VCV**	**VV**
Normal (%)	87.6 ± 13.4	89.4 ± 6.9	95.9 ± 3.2^#^	55.6 ± 4.9^*^	74.8 ± 9.2^**^, ^#^	91.2 ± 3.1^**^,^‡^
Collapse (%)	5.3 ± 3.9	5.9 ± 5.3	4.0 ± 3.2	4.7 ± 1.7	11.2 ± 6.8^**^	5.3 ± 2.6^‡^
Hyperinflation (%)	2.5 ± 7.0	4.7 ± 5.1	0.0 ± 0.0	39.7 ± 4.9^*^	14.0 ± 7.6^**^, ^#^	3.5 ± 2.6^**^,^‡^
Lm (μ m)	48.2 ± 8.8	64.2 ± 23.4	69.6 ± 18.9	66.3 ± 10.8^*^	104.9 ± 17.1^**^, ^#^	97.6 ± 16.7^**^,^†^
EELV (mL)	3.2 ± 0.8	2.4 ± 0.5	2.6 ± 0.8	4.6 ± 1.2^*^	3.7 ± 0.9^#^	3.7 ± 1.2^†^

Values are mean ± standard deviation (SD) of eight animals in each group. SAL: animals that received saline and were analyzed 5 weeks after the last instillation; ELA: animals that received elastase and were analyzed 5 weeks after the last elastase endotracheal instillation; NV: non-ventilated; VCV: volume-controlled ventilation; VV: variable ventilation. Normal: normal fraction area as percentage; Collapse: collapsed fraction area as percentage; Hyperinflation: hyperinflated fraction area as percentage; Lm: mean linear intercept; EELV: End-expiratory lung volume. Comparisons were performed by two-way repeated-measures ANOVA followed by Bonferroni's post-hoc test (p < 0.05).

* Significantly different from SAL-NV (p < 0.05).

** Significantly different from ELA-NV (p < 0.05).

# Significantly different from SAL-VCV (p < 0.05).

‡ Significantly different from ELA-VCV (p < 0.05).

† Significantly different from SAL-VV (p < 0.05).

In the SAL group, the PAT/PET ratio was higher in VV compared to VCV (VV, 0.62 ± 0.12 vs. VCV, 0.42 ± 0.12; *p* = 0.023), but in the ELA group, no differences were observed between VV and VCV (Table 3). In the ELA group, RV area was larger in VV than VCV (VV, 0.42 ± 0.07 vs. VCV, 0.31 ± 0.01; *p* = 0.039).

**Table 3:** **Echocardiography data at Baseline PEEP and End**.

		**SAL**		**ELA**	
		**VCV**	**VV**	**VCV**	**VV**
RV area (mm^2^)	Baseline PEEP	0.25 ± 0.04	0.25 ± 0.07	0.38 ± 0.07^#^	0.39 ± 0.08^†^
	End	0.34 ± 0.14	0.28 ± 0.05	0.31 ± 0.01	0.42 ± 0.07^‡^, ^†^
PAT/PET	Baseline PEEP	0.51 ± 0.05	0.51 ± 0.07	0.41 ± 0.04^#^	0.42 ± 0.04^†^
	End	0.42 ± 0.12	0.62 ± 0.12^#^, ^&^	0.44 ± 0.09	0.37 ± 0.09^†^
LV area (mm^2^)	Baseline PEEP	0.17 ± 0.04	0.14 ± 0.07	0.18 ± 0.02	0.17 ± 0.08
	End	0.19 ± 0.09	0.16 ± 0.06	0.19 ± 0.03	0.21 ± 0.07
EF (%)	Baseline PEEP	91.3 ± 2.7	94.3 ± 4.7	93.2 ± 5.2	90.3 ± 6.8
	End	90.2 ± 4.6	92.0 ± 4.3	89.2 ± 4.6	91.6 ± 2.2
FS (%)	Baseline PEEP	57.9 ± 4.0	66.0 ± 11.5	62.3 ± 9.0	57.8 ± 10.5
	End	56.8 ± 7.0	59.6 ± 7.2	52.5 ± 7.1	58.2 ± 3.6
HR (bpm)	Baseline PEEP	387 ± 43	404 ± 39	439 ± 30	386 ± 42
	End	388 ± 41	411 ± 31	410 ± 53	375 ± 51
IVC diameter (mm)	Baseline PEEP	0.22 ± 0.04	0.23 ± 0.07	0.20 ± 0.04	0.18 ± 0.03
	End	0.22 ± 0.03	0.26 ± 0.03	0.22 ± 0.06	0.27 ± 0.03^&^
RA diameter (mm)	Baseline PEEP	0.42 ± 0.06	0.46 ± 0.05	0.42 ± 0.05	0.42 ± 0.08
	End	0.48 ± 0.10	0.47 ± 0.12	0.44 ± 0.09	0.52 ± 0.11

Values are mean ± standard deviation (SD) of eight animals in each group. SAL: animals that received saline and were analyzed 5 weeks after the last instillation; ELA: animals that received elastase and were analyzed 5 weeks after the last elastase endotracheal instillation; VCV: volume-controlled ventilation; VV: variable ventilation; RV area: right ventricular end-diastolic area; PAT/PET: relation between pulmonary artery systolic acceleration time (PAT) and pulmonary artery systolic ejection time (PET) on pulsed-wave Doppler; LV: left ventricular end-diastolic area; EF: ejection fraction; FS: fractional shortening; HR: heart rate; IVC: inferior vena cava; RA: right atrium. Comparisons were performed by two-way repeated-measures ANOVA followed by Bonferroni's post-hoc test (p < 0.05). Paired t-tests were done to compare Baseline PEEP and End.

# Significantly different from SAL-VCV (p < 0.05).

† Significantly different from SAL-VV (p < 0.05).

‡ Significantly different from ELA-VCV (p < 0.05).

& Significantly different from Baseline PEEP (p < 0.05).

**Figure 3:**
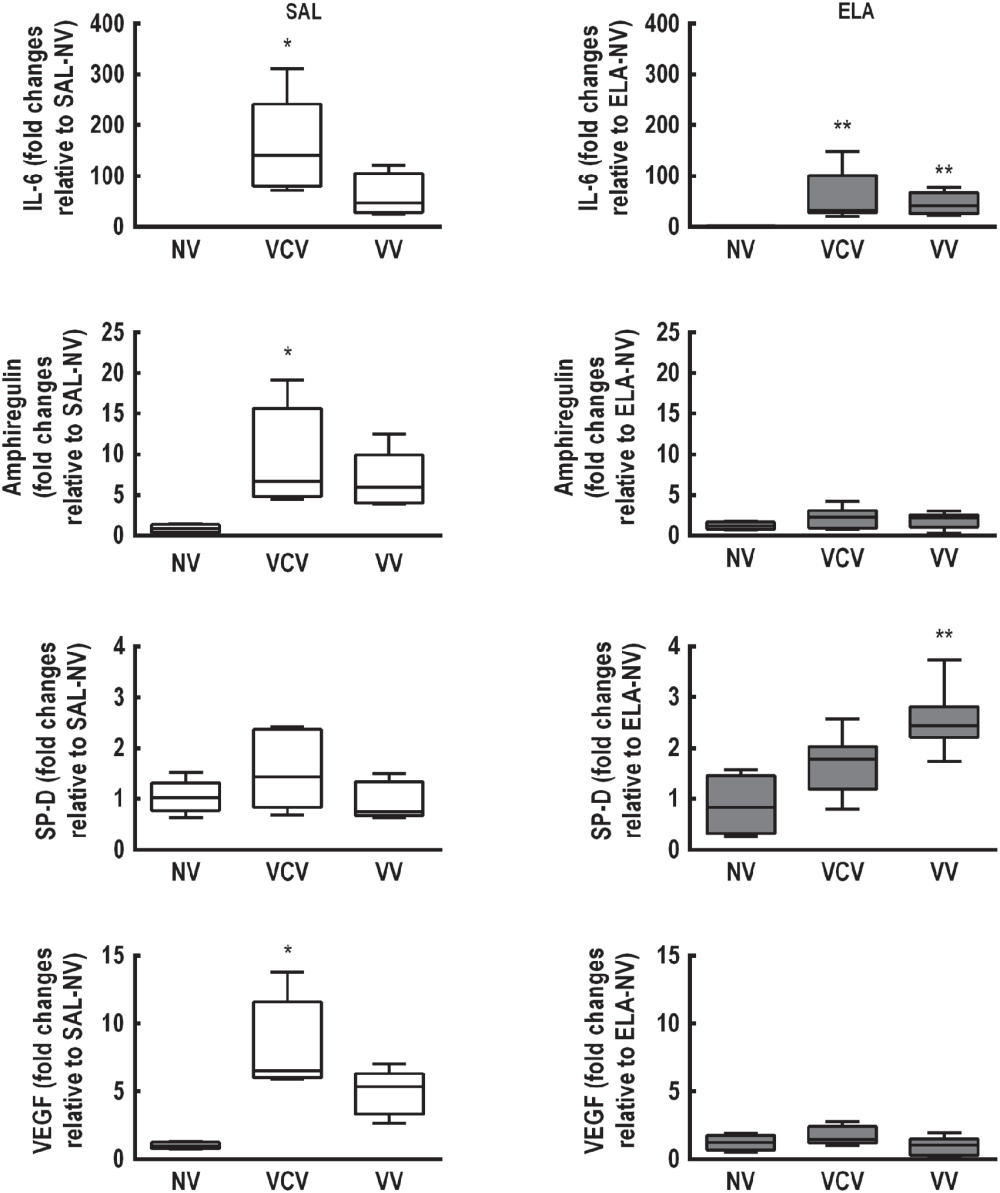
**Real-time polymerase chain reaction analysis of biological markers associated with inflammation [interleukin (IL)-6], alveolar stretch (amphiregulin), type II epithelial cell mechanotransduction [surfactant protein (SP)-D], and endothelial injury [vascular endothelial growth factor (VEGF)]**. SAL: animals that received saline and were analyzed 5 weeks after the last saline endotracheal instillation; ELA: animals that received elastase and were analyzed 5 weeks after the last elastase endotracheal instillation. Data are presented as box plots of median and interquartile range (whiskers indicate the 10th and 90th percentiles), and refer to eight animals in the SAL and ELA groups. Relative gene expression was calculated as the ratio of average gene expression compared with the reference gene (36B4) and expressed as fold change relative to SAL-NV or ELA-NV, as appropriate. *Significantly different from SAL-NV group (*p* < 0.05). **Significantly different from ELA-NV group (*p* < 0.05).

In the SAL group, mRNA expressions of IL-6, amphiregulin, and VEGF were higher in VCV compared to NV [IL-6: VCV, 140.5 (79.9–241.7) vs. NV, 0.64 (0.62–0.88), *p* = 0.004; amphiregulin: VCV, 6.7 (4.8–15.7) vs. NV, 0.81 (0.36–1.38), *p* = 0.012; VEGF: VCV, 6.53 (5.98–11.58) vs. NV, 0.95 (0.76–1.25), *p* = 0.003], irrespective of mechanical ventilation mode (Figure 3). In the ELA group, IL-6 expression was higher in both VCV [31.46 (27.52–100.9)] and VV [40.93 (25.37–66.95)] compared to NV [0.47 (0.21–1.74), *p* = 0.021 and *p* = 0.013 respectively]. VV animals exhibited increased SP-D expression compared to NV animals (Figure 3) [VV, 2.44 (2.21–2.82) vs. NV, 0.84 (0.32–1.46), *p* = 0.008].

## Discussion

In the first protocol, biological markers associated with inflammation, alveolar stretch, and damage inflicted to alveolar endothelial cells were increased at the time point of greatest cardiorespiratory impairment [high specific lung elastance and large right ventricular end-diastolic area (5 weeks after the last elastase endotracheal instillation)], thus characterizing a successful model of elastase-induced emphysema. In the second protocol, variable ventilation (compared to conventional volume-controlled ventilation) reduced respiratory system elastance, lung hyperinflation, and alveolar collapse, and augmented surfactant protein-D expression; however, it also increased right ventricular end-diastolic area.

### First protocol: characterization of elastase-induced emphysema in rats

In the model of elastase induced-emphysema used herein, lung mechanics, morphology, fibrogenesis, and elastolysis were altered at different time points. At 5 weeks, most of these changes were associated with lower ratio between PAT and PET, enlarged right ventricular end-diastolic area, and increased mRNA expression of interleukin-6, cytokine-induced neutrophil chemoattractant, amphiregulin, angiopoietin-2, and vascular endothelial growth factor compared to SAL. In addition, according to CT measurements, ELA animals showed lung attenuation coefficients similar to those observed in other models of elastase-induced pulmonary emphysema (Onclinx et al., 2006) and in human emphysema, with lung CT voxels exhibiting attenuation values in the region of −950 HU (low attenuation areas) (Bhavani et al., 2015). Our model differs from several others of elastase-induced emphysema due to animal strain (Onclinx et al., 2006; Schmiedl et al., 2008; Tolnai et al., 2012; Kawago et al., 2014) and elastase dose and regimen (Onclinx et al., 2006; Schmiedl et al., 2008; Di Petta et al., 2011; Tolnai et al., 2012); most use a single dose (Schmiedl et al., 2008; Di Petta et al., 2011; Tolnai et al., 2012), involve evaluation at only one time point, and do not focus on the extrapulmonary impairment which is usually present in severe human emphysema. Yokoyama et al. (1987) found morphological changes characteristic of emphysematous lesions 7–10 weeks after single intratracheal administration of elastase (6.5 units) in rats. Furthermore, recent studies in mice (Cruz et al., 2012; Antunes et al., 2014) reported that multiple elastase instillations can lead to cardiorespiratory alterations.

### Second protocol: comparison between variable and conventional volume-controlled ventilation

Previous experimental studies (Spieth et al., 2009b; Thammanomai et al., 2013) have shown that VV improves gas exchange and respiratory mechanics, as well as reduces lung damage and inflammation in experimental ARDS. To the best of our knowledge, the present study was the first to investigate the effects of VV and VCV on lung morphofunction and biological markers associated with VILI in experimental emphysema. To minimize the potential effects of VV and VCV, a protective tidal volume was used during both strategies.

#### Respiratory system mechanics and lung histology

The fact that VV, but not VCV, decreased dynamic respiratory system elastance and non-linearity index of the dynamic respiratory system elastance over time is likely explained by lung recruitment (Kano et al., 1994; Bersten, 1998). Alveolar collapse and hyperinflation were lower in VV compared to VCV, which may have led to better distribution of regional ventilation and of stress and strain across the lungs. Our observation that VV did not improve gas exchange compared to VCV might be explained by differences in regional perfusion (Pelosi and de Abreu, 2015).

Variable ventilation reduced alveolar collapse and hyperinflation only in ELA thus resulting in decreased dynamic respiratory system elastance and volume-dependent elastance. We hypothesize that VV promoted alveolar recruitment by periodic higher inspiratory pressures, reducing the traction of alveolar walls by collapsed regions and thus mitigating hyperinflation. In short, VV resulted in better distribution of regional ventilation, stress, and strain across the lungs.

#### Cardiac impairment

In SAL animals, after VV, the fraction area of normal alveoli increased, thus resulting in a reduction of pulmonary vascular resistance, as shown by the increase in the ratio between pulmonary artery systolic acceleration time and pulmonary artery systolic ejection time. This is expected in a normal capillary density scenario (Overholser et al., 1994); however, similar behavior was not observed in ELA animals. After VV, even though the fraction area of normal alveoli increased, pulmonary vascular resistance may have increased as well, thus leading to enlargement of right ventricular end-diastolic area. The relationship between pulmonary vascular resistance and lung volume is “U-shaped,” where the lowest pulmonary vascular resistance values are closer to functional residual capacity (Simmons et al., 1961). In elastase-induced emphysema, arterial remodeling occurs, with increased elastolysis in the vessel wall (Wills et al., 1996). Furthermore, hyperinflated and collapsed alveoli may not receive adequate blood flow, due to vascular compression and hypoxic vasoconstriction, respectively; thus, blood flow could be driven toward normal alveoli. After VV, there was likely a better airflow distribution across the airways, which may have led to improved lung volume distribution, since there was no change in total lung volume as detected by EELV measurements. We hypothesized that, after lung volume redistribution, a reduction in alveolar hyperinflation and recruitment of alveolar collapse occurred, alongside a slight decrease in size of normal alveoli, resulting in increased pulmonary vascular resistance, as indirectly measured by echocardiographic analysis (ratio between pulmonary artery systolic acceleration time and pulmonary artery systolic ejection time, and right ventricular end-diastolic area). This effect can move the point from the lowest pulmonary vascular resistance at functional residual capacity to the right side of the curve, thus increasing the pulmonary vascular resistance by alveolar vessel distortion. Additionally, during VV, the higher frequency of increased tidal volume and plateau pressure in a fixed time course may lead to increased pulmonary vascular resistance, especially in the presence of vessel wall changes.

#### Biological markers

In SAL animals, VCV, but not VV, led to increased mRNA expression of interleukin-6, amphiregulin, and vascular endothelial growth factor compared to NV. In this line, an *in vitro* study reported that exposure of normal cultured epithelial cells to increased stress and strain results in cell membrane disruption and death (Tschumperlin and Margulies, 1999). In non-injured lungs, VV reduced stress and strain, preventing inflammation, alveolar stretch, and endothelial damage. In ELA, no difference was observed between VV and VCV regarding interleukin-6 mRNA expression, suggesting that mechanical ventilation *per se* induces activation of lung inflammation. In the emphysematous lung, which tends to be more structurally fragile (Suki et al., 2013), moderate and high tidal volumes (based on the normal distribution with a 30% coefficient of variation) during VV could increase lung inflammation. On the other hand, VV resulted in beneficial biological effects through an increase in surfactant protein-D expression, which is in agreement with previous experimental findings in experimental ARDS (Thammanomai et al., 2013). We may speculate that application of variable stretch to alveolar epithelial cells led to mechanotransduction toward intracellular signaling and cytoplasmic reorganization in an attempt to restore surfactant production (Roan and Waters, 2011).

## Limitations

This study has several limitations which must be taken into account. First, emphysema was induced by intratracheal administration of elastase, and our results cannot be extended to other emphysema models with different degrees of severity, nor to human emphysema. Second, the PEEP levels used in the current study, while often used in rats, may not be directly extrapolated to the clinical setting. Third, the coefficient of variation of tidal volume was based on existing acute lung injury models (Spieth et al., 2009a) that showed 30% as the best compromise between lung mechanics and blood gas exchange, since no study of VV in emphysema has been conducted. This degree of variation resulted in additional right ventricular load. Thus, future studies evaluating different tidal volume coefficients of variation in experimental emphysema are warranted. Fourth, echocardiographic images were not gated to respiratory period or tidal volume, which may have affected cardiac parameter measurements. However, echocardiographic images were collected during 15 min at each time point, which may have minimized potential bias. Finally, mediators were measured in lung tissue, but not in blood, and the observation time was relatively short, thus precluding assessment of potential changes in protein levels of all biological markers of interest; keeping animals with emphysema hemodynamically stable for more than 6 h to measure actual protein levels would have required administration of greater amounts of fluids, which could have interfered with gene expression. Even though no emphysema model is able to reproduce all features of human emphysema, the rat model of elastase-induced emphysema used in this study induces lung morphological changes that may provide a more efficient tool to better understand the cardiorespiratory effects after mechanical ventilation, with potential for translation into clinical practice. This is a first step toward understanding the mechanism of VILI in emphysema.

In conclusion, compared to VCV, VV improved lung mechanics and histology as well as augmented surfactant protein-D gene expression, but increased right ventricular end-diastolic area in a rat model of elastase induced-emphysema.

## Author contributions

Conceived and designed the experiments: IH, GP, RH, RG, RL, PP, MD, PS, PR. Performed experiments: IH, GP, RH, CW, PM, IR NR, FC, RS, MD, SS, PS, PR. Analyzed data: IH, GP, RH, CW, PM, IR NR, FC, RS, MD, SS, PS, PR. Interpreted results of research: IH, GP, RH, RG, RL, PP, MD, PS, PR. Drafted, edited, critically revised paper: IH, GP, RH, NR, PP, MGD, PS, PR. All authors approved final version of manuscript.

## Funding

This study was supported by the Brazilian Council for Scientific and Technological Development (CNPq), the Rio de Janeiro State Research Foundation (FAPERJ), the Department of Science and Technology (DECIT)/Brazilian Ministry of Health, and the Coordination for the Improvement of Higher Education Personnel (CAPES).

### Conflict of interest statement

The authors declare that the research was conducted in the absence of any commercial or financial relationships that could be construed as a potential conflict of interest.

## Footnotes

^1^Global Initiative for Obstructive Lung Disease. Global Strategy for the Diagnosis, Management and Prevention of COPD, Global Initiative for Chronic Obstructive Lung Disease (GOLD) 2015. Available online at: http://www.goldcopd.org/
