# Supplementary material for: Comparison between Variable and Conventional Volume-Controlled Ventilation on Cardiorespiratory Parameters in Experimental Emphysema
Source: Front Physiol. 2016 Jun 30;7:277. doi: 10.3389/fphys.2016.00277 (PMC4928149; doi:10.3389/fphys.2016.00277)
Supplement: Supplementary file 1 [file Presentation1.pdf]

Supplementary Material

Comparison between variable and conventional volume-controlled ventilation on cardiorespiratory parameters in experimental emphysema

Isabela Henriques[1#], Gisele A Padilha[1#], Robert Huhle[2#], Caio Wierzchon[1], Paulo JB Miranda[1], Isalira P Ramos[3,4], Nazareth Rocha[1,5], Fernanda F Cruz[1], Raquel S Santos[1], Milena Vasconcellos de Oliveira[1], Sergio A Souza[4,6], Regina C Goldenberg[3], Ronir R Luiz[7], Paolo Pelosi[8], Marcelo Gama de Abreu[2], Pedro L Silva[1], Patricia R M Rocco[1]

1. Laboratory of Pulmonary Investigation, Carlos Chagas Filho Biophysics Institute, Federal University of Rio de Janeiro, Brazil
2. Pulmonary Engineering Group, Department of Anesthesiology and Intensive Care Therapy, University Hospital Carl Gustav Carus, Technische Universität Dresden, Germany
3. Laboratory of Molecular and Cellular Cardiology, Carlos Chagas Filho Biophysics Institute , Federal University of Rio de Janeiro, Brazil
4. *National Center* for Structural *Biology* and Bioimaging (*CENABIO*), Federal University of Rio de Janeiro, Brazil
5. Department of Physiology and Pharmacology, Biomedical Institute, Fluminense Federal University, Brazil
6. Nuclear Medicine Service, Clementino Fraga Filho University Hospital, Federal University of Rio de Janeiro, Rio de Janeiro, Brazil.
7. Institute of Public Health Studies, Federal University of Rio de Janeiro, Rio de Janeiro, Brazil
8. Department of Surgical Sciences and Integrated Diagnostics, IRCCS AOU San Martino IST, University of Genoa, Genoa, Italy

ADDITIONAL METHODS

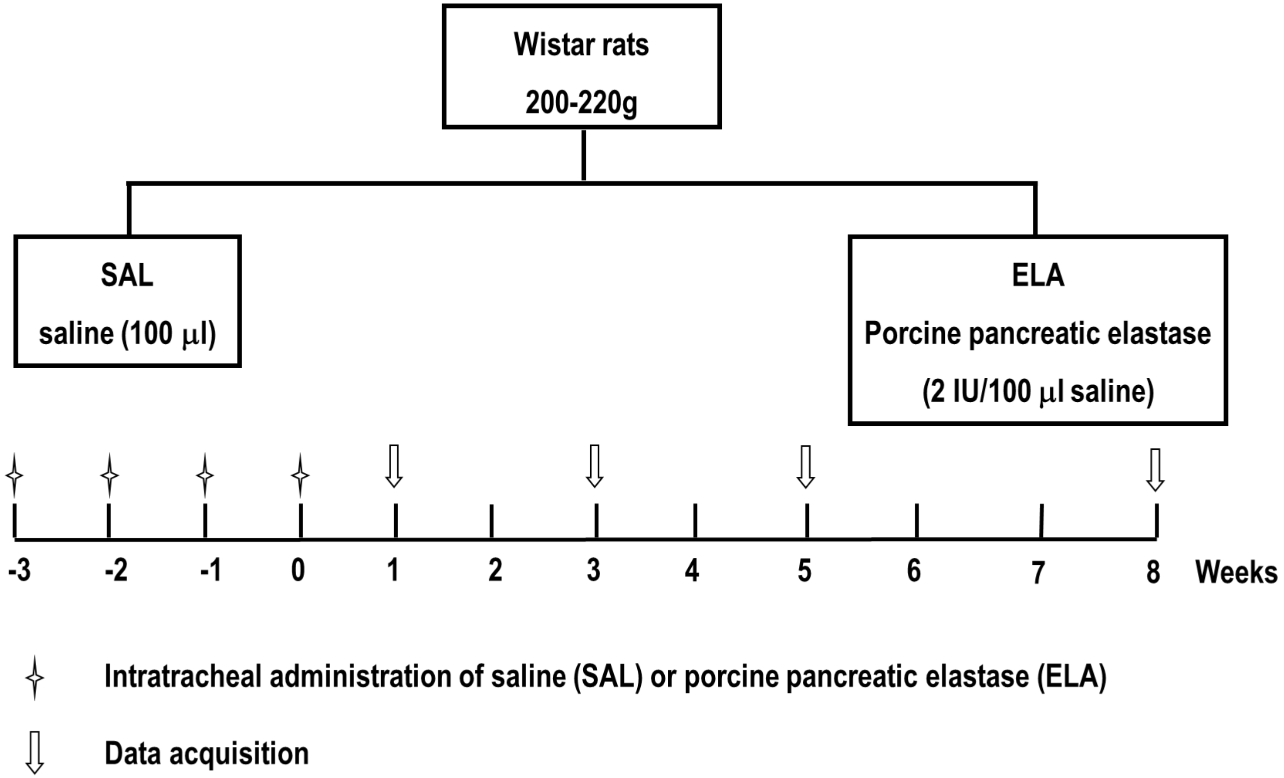

Figure S1. Experimental design for model characterization. SAL, intratracheal instillation of saline; ELA, intratracheal instillation of porcine pancreatic elastase.

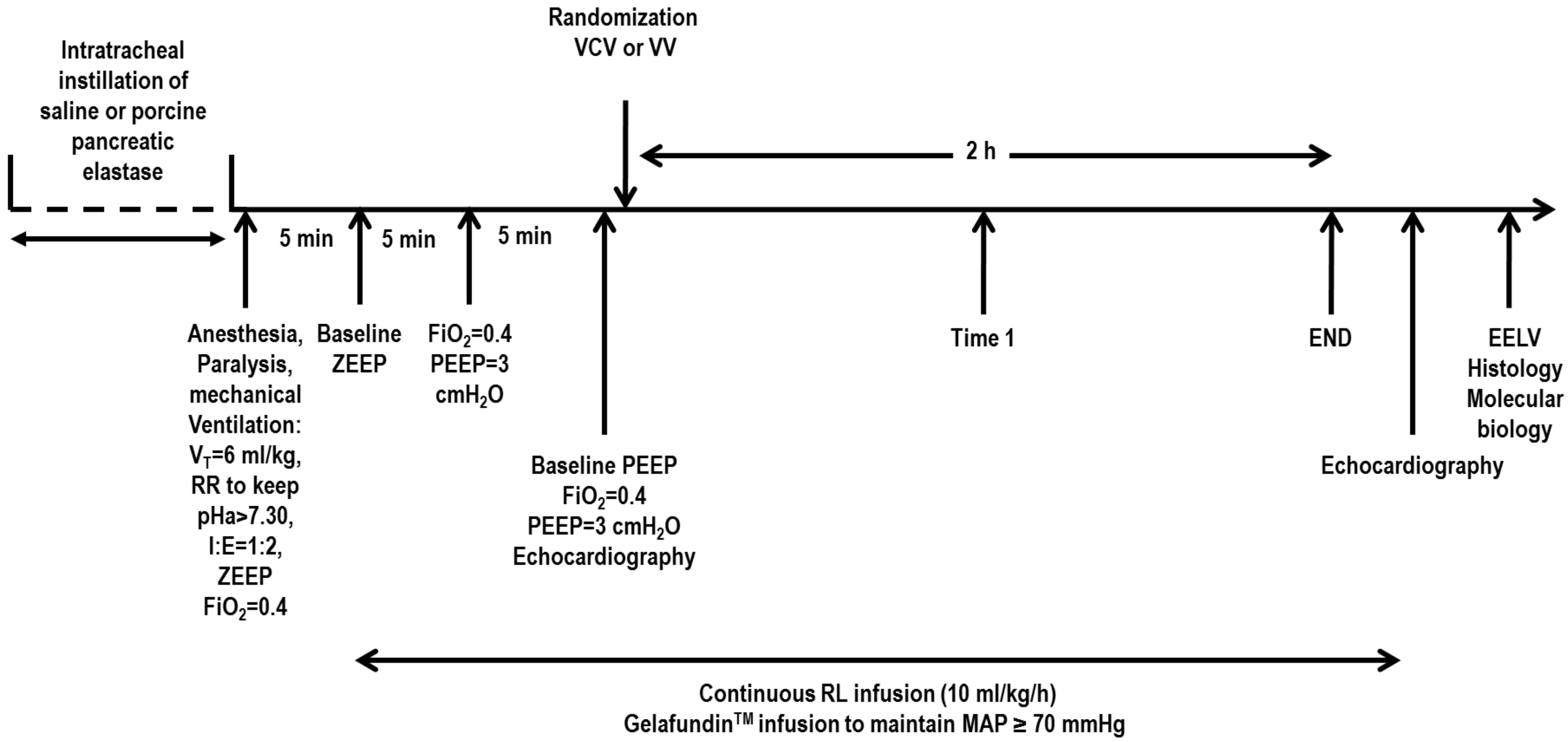

Figure S2. Experimental timeline. V_T, tidal volume; RR, respiratory rate; pHa: arterial pH; I:E, inspiratory-to-expiratory ratio; PEEP, positive end-expiratory pressure; ZEEP, zero end-expiratory pressure; F_{IO_2}, fraction of inspired oxygen; RL, Ringer's lactate; MAP, mean arterial pressure; VCV, conventional volume-controlled ventilation; VV, variable ventilation; Time 1: 1 hour mechanical ventilation; END: 2 hour mechanical ventilation; EELV: end-expiratory lung volume.

Table S1. Forward and reverse oligonucleotide sequences of target gene primers

Gene	Primer	Primer sequences (5′-3′)
IL-6	Forward	CTC CGC AAG AGA CTT CCA G
	Reverse	CTC CTC TCC GGA CTT GTG A
CINC-1	Forward	TGC ACC CAA ACC GAA GTC AT
	Reverse	TTG TCA GAA GCC AGC GTT CAC
SP-D	Forward	AAATCTTCAGGGCGGCAAA
	Reverse	GGCCTGCCTGCACATCTC
Amphiregulin	Forward	TTTCGCTGGCGCTCTCA
	Reverse	TTCCAACCCAGCTGCATAATG
Ang-2	Forward	CAGCCAACCAGGTGATT
	Reverse	AAGTTGGAAGGACCACATGC
VEGF	Forward	CAG AAA GCC CAT GAA GTG GT
	Reverse	ACA CAG GAC GGC TTG AAG AT
36B4	Forward	AAT CCT GAG CGA TGT GCA G
	Reverse	GCT GCC ATT GTC AAA CAC

Primers used in experiments. IL-6, interleukin-6; CINC-1, cytokine-induced neutrophil chemoattractant 1; SP-D, surfactant protein D; Ang-2, angiopoietin-2; VEGF, vascular endothelial growth factor; 36B4, acidic ribosomal phosphoprotein P0.

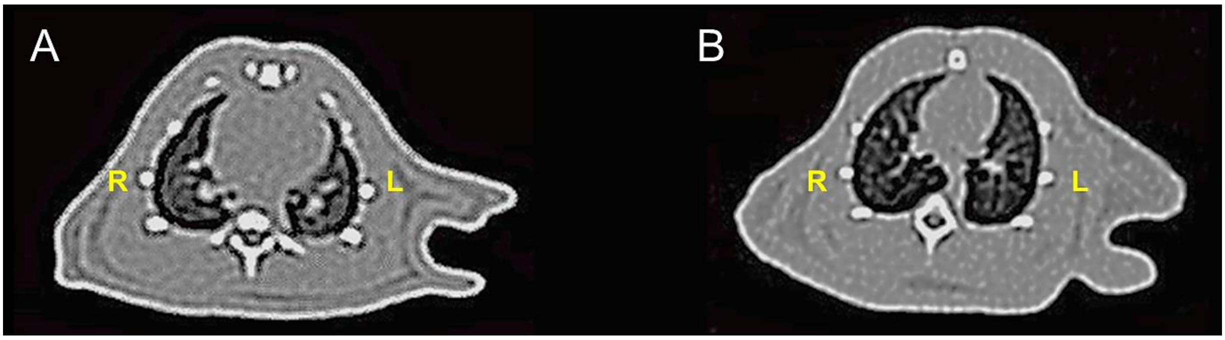

Figure S3. A. SAL animal. B. ELA animal. R, right lung; L, left lung. Analysis of Hounsfield units (HU) in the SAL animal: right lung, -543 HU (minimum) to -496 HU (maximum); left lung, -608 HU (minimum) to -525 HU (maximum). Analysis of HU in the ELA animal: right lung, -914 HU (minimum) to -466 HU (maximum); left lung, -929 HU (minimum) to -842 HU (maximum).

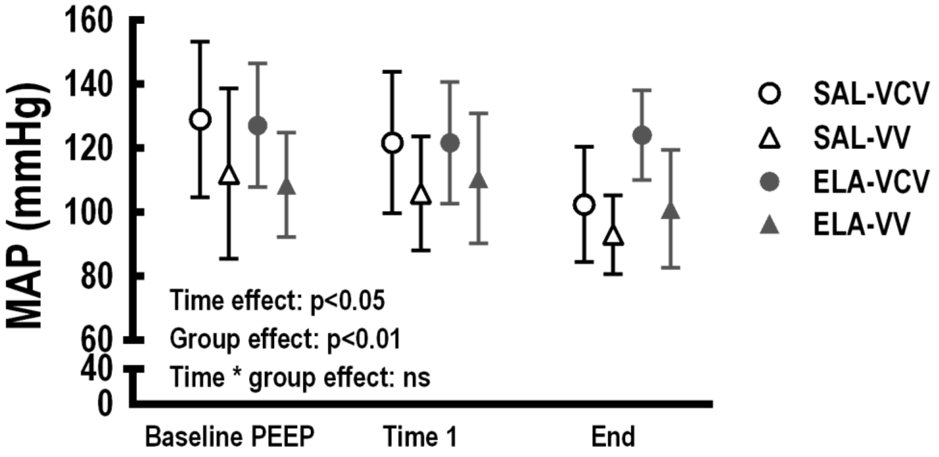

Figure S4. Mean arterial pressure (MAP) over time. Measurements were obtained at Baseline-PEEP, Time 1 (1 hour mechanical ventilation), and End (2 hours mechanical ventilation). Symbols represent the mean ± standard deviation (SD) of 8 animals per group. SAL: animals that received saline and were analyzed 5 weeks after the last saline endotracheal instillation; ELA: animals that received elastase and were analyzed 5 weeks after the last elastase endotracheal instillation; VCV, conventional volume-controlled ventilation; VV, variable ventilation. Comparisons among groups (group effect), over time (time effects), and their interaction (time × group effect) were performed by means of two-way repeated-measures ANOVA followed by Bonferroni's post-hoc test ($p<0.05$).

Table S2. Respiratory variables and blood gas analysis at Baseline ZEEP

	SAL		ELA	
	VCV	VV	VCV	VV
V_T (mL/kg)	6.0 ± 0.1	6.0 ± 0.1	5.9 ± 0.1	6.0 ± 0.3
CV of V_T (%)	1.6 ± 0.4	1.7 ± 1.1	1.3 ± 0.7	2.1 ± 1.4
V'_E ($mL.min^{-1}$)	150.1 ± 1.8	148.0 ± 2.6	147.4 ± 2.0	147.4 ± 2.0
$E,_{RS}$ (cmH_2O/mL)	3.9 ± 0.6	3.7 ± 0.3	4.0 ± 0.8	3.9 ± 0.3
$E1,_{RS}$ (cmH_2O/mL)	3.7 ± 1.2	3.7 ± 0.9	3.9 ± 1.3	3.5 ± 0.8
$E2,_{RS}$ (cmH_2O/mL)	0.13 ± 0.33	0.20 ± 0.26	0.55 ± 0.52	0.18 ± 0.46
R ($cmH_2O/mL/s$)	3.7 ± 0.6	3.7 ± 0.9	2.9 ± 1.3	3.5 ± 0.8
PEEPi (cmH_2O)	0.4 ± 0.0	0.4 ± 0.1	0.4 ± 0.1	0.4 ± 0.1
pHa	7.43 ± 0.06	7.41 ± 0.02	7.37 ± 0.10	7.40 ± 0.06
$PaCO_2$ (mmHg)	34 ± 5	36 ± 4	41 ± 7	41 ± 4
PaO_2 (mmHg)	97 ± 19	89 ± 30	82 ± 22	108 ± 27
HCO_3^- (mEq/L)	24 ± 2	23 ± 2	23 ± 4	25 ± 3

SAL: animals that received saline and were analyzed 5 weeks after the last saline endotracheal instillation; ELA: animals that received elastase and were analyzed 5 weeks after the last elastase endotracheal instillation; VCV, volume-controlled ventilation; VV, variable ventilation; V_T, tidal volume; CV of V_T, coefficient of variation of tidal volume; V'_E, minute ventilation; $E,_{RS}$, dynamic lung elastance; $E1,_{RS}$, volume-independent elastance; $E2,_{RS}$, volume-dependent elastance; R, airway resistance; PEEPi, intrinsic positive end-expiratory pressure; pHa, arterial pH; $PaCO_2$, arterial carbon dioxide partial pressure; PaO_2, arterial oxygen partial pressure; HCO_3^- bicarbonate. Values are given as mean ± standard deviation of 8 animals/group.
